# The College of Nursing and Affiliated Training Schools

**Published:** 1920-12-04

**Authors:** 


### The College of Nursing and Affiliated Training Schools.

The College of Nursing has always concerned itself very sympathetically with the position of small and special hospitals. The difficulties experienced in staffing them is well known, but the College has been more concerned to find some method by which the excellent experience obtained in these hospitals may be made to contribute to the full general training. To effect this hospitals have been encouraged to draft schemes of affiliation with training-schools, which, on being submitted to the Council, if approved, have been put into operation. There were, previous to the existence of the College, affiliated schools affording excellent training. These schools, however, had never been recognised by the Central Midwives Board for the purpose of the shortened course of training for trained nurses, namely, four months instead of six.

The College has taken the matter up with the Board, asking that nurses holding the certificate of affiliated schools recognised by the College may have the privilege of the four months' training. The result was announced in last week's Press.

There are, however, two points to bring before members of the College, the first being that the new conditions cannot take effect until July, as the rules have to be revised; and, secondly, that members must not confuse this affiliated training with the recognition of individual small hospitals having a daily average of less than a hundred beds, which is the standard set by the Central Midwives Board. In this connection it must be remembered that the regulations of the College for admission to its Register are as during a period of grace, and the standard has yet to be set subsequent to that period.

The Council of the College the week before last received a deputation of matrons from different parts of England to discuss with them the problems of the smaller training-schools. The deputation was introduced by Miss Ind, Matron of The Hospital, Stratford-on-Avon, who, it will be remembered, read a most interesting paper on the subject at a nursing conference in June last. The Council was delighted to have this opportunity of conferring with matrons who have experienced the difficulties of administration of small hospitals, and it is hoped, as a result, that these hospitals will be utilised in a scheme of general training.

